# Factors Associated with Poststroke Fatigue: A Systematic Review

**DOI:** 10.1155/2015/347920

**Published:** 2015-05-25

**Authors:** Amélie Ponchel, Stéphanie Bombois, Régis Bordet, Hilde Hénon

**Affiliations:** ^1^Department of Pharmacology, University of Lille, INSERM U1171, 59045 Lille, France; ^2^Department of Neurology, Stroke Center, University of Lille, INSERM U1171, 59045 Lille, France; ^3^Department of Neurology, Memory Clinic, University of Lille, INSERM U1171, 59045 Lille, France

## Abstract

*Background.* Poststroke fatigue (PSF) is a frequent, disabling symptom that lacks a consensual definition and a standardized evaluation method. The (multiple) causes of PSF have not been formally characterized. *Objective.* To identify factors associated with PSF. *Method.* A systematic review of articles referenced in MEDLINE. Only original studies having measured PSF and potentially associated factors were included. Data was extracted from articles using predefined data fields. *Results.* Although PSF tends to be more frequent in female patients and older patients, sociodemographic factors do not appear to have a major impact. There are strong associations between PSF and emotional disturbances (such as depression and anxiety). PSF may also be linked to attentional disturbances (mainly slowing in processing speed). The literature data have failed to demonstrate a clear impact of the type and severity of stroke. It has been suggested that PSF results from alterations in the frontothalamostriatal system and/or inflammatory processes. Pain, sleep disorders, and prestroke fatigue also appeared to be associated with PSF. *Implications.* A better understanding of PSF may improve stroke patient care and facilitate the development of effective treatments.

## 1. Introduction

Stroke is the second-ranked cause of mortality in the world and a major cause of disability [1]. Whether ischemic or hemorrhagic, stroke can induce physical disabilities [2] and cognitive, psychological, and behavioral impairments [3]. Poststroke depression is of particular interest [4]. For several decades, fatigue was considered to be a symptom of poststroke depression. However, the fact that depression-free patients frequently complain of fatigue has prompted the examination of “poststroke fatigue” (PSF) as a specific syndrome [5].

At present, there is no consensual, clear definition of PSF and this is partly because of the syndrome's complexity. PSF differs from normal fatigue [6–9] that results from overexertion and is relieved by rest [10]. In fact, PSF is a disease state characterized by a chronic, persistent, excessive lack of energy [10–12] with an impact on activities of daily living [13]. PSF is generally defined in subjective terms as an overall state of feeling: “*a feeling of early exhaustion, weariness, and aversion to effort*” [14]. This type of fatigue has been studied with qualitative approaches such as patient interviews [15] and patient questionnaires like the Fatigue Severity Scale (FSS) [16, 17].

Hence, the wide range of PSF prevalence values found in the literature (from 16% [18] up to 74% [19] of patients) may be due to the variety of tools used to measure PSF (based on either unidimensional or multidimensional conceptual frameworks of fatigue) [12], the lack of a consensual definition, and the heterogeneity of stroke patients in terms of age, stroke type and severity, and comorbidities [11].

Besides being frequent, PSF was judged by between 23% and 59.5% of stroke patients to be one of their worst symptoms [5, 20–25]. Although fatigue is thought to be less severe and less specific after stroke than in multiple sclerosis, it seems to have similar functional impacts on psychological functioning and professional, social, and familial activities [26]. Furthermore, PSF has a negative impact on subjective feelings of recovery during rehabilitation [27].

PSF is a major cause of “invisible” handicap because levels of awareness of this condition among relatives, work colleagues, and even medical staff are low. Two studies have shown that patients receive little information about PSF [7, 28] and do not understand why they feel fatigued [29]. In turn, this leads to difficulties in coping with fatigue and then anxiety, depression, guilt, and a fall in self-esteem [28]. Fatigue may also lead to misunderstanding of the patient's behavior by his/her family or friends; excessive demands may exceed the patient's abilities, maintain anxiety or depression, and lead to withdrawal from certain activities and social life [7].

PSF probably results from complex, poorly understood interactions between biological, psychosocial, and behavioral phenomena. We consider that it is important to try to understand PSF more deeply and thus identify at-risk patients and develop novel treatments. Here, we performed a systematic review of studies of PSF, with a particular focus on associated factors (whether sociodemographic, psychocognitive, or neurophysiological).

## 2. Methods

### 2.1. Search Strategy

We systematically searched the MEDLINE database via PubMed (up to May 5, 2015) using logical combinations of the keywords “fatigue,” “tiredness,” or “exhaustion” with “stroke,” “transient ischemic attack” (TIA), “intracranial hemorrhage,” or “subarachnoid hemorrhage.” We did not apply any time or language limitations.

### 2.2. Eligibility Criteria

We included only original, observational studies of individuals with stroke (whether first or recurrent, ischemic or hemorrhagic). The studies had to assess PSF (using a single question, a case definition or a fatigue scale) and at least one factor associated with PSF (either as a dichotomous or continuous variable).

Studies were excluded if they (i) lacked primary data (i.e., review articles, editorials, or protocol papers), (ii) were case studies, (iii) did not distinguish between data on stroke patients and data on other participants, (iv) did not quantitatively assess PSF or only recorded physical parameters (e.g., electromyography), and (v) contained no data for a valid analysis of putative associations between PSF and other factors.

**Figure 1:**
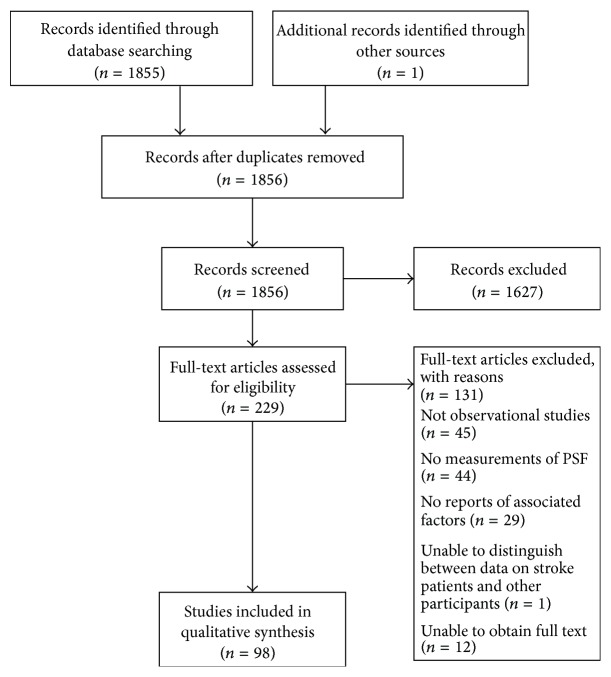
Study flow diagram.

Our PubMed search identified 1855 individual records. Firstly, all titles and abstracts were screened for eligibility. We identified 1627 articles that did not match our criteria in terms of the study population (i.e., not stroke patients) and the article format (review articles, editorials, protocol papers, and case studies), leaving 228 relevant studies. The reference lists of retrieved articles were checked for other potentially relevant studies, and one other paper was identified as eligible. Hence, 229 articles were assessed for eligibility. Twelve of these articles could not be obtained, and so 217 full texts of potentially eligible publications were retrieved and read. Forty-five papers were excluded because they were not observational studies (19 were interventional trials and 26 were review articles). Analysis of the Methods sections enabled the exclusion of 44 studies because fatigue had not been assessed quantitatively. Twenty-nine papers did not provide data concerning putative associations between PSF and other factors, and one did not distinguish between data on stroke patients and data on other participants. Thus, 98 papers met our inclusion criteria and were included in the review (Figure 1, constructed in accordance with the Preferred Reporting Items for Systematic Reviews and Meta-Analyses (PRISMA) statement [30]).

### 2.3. Data Extraction

We extracted data on study characteristics (the sample size and the methods used to assess PSF), participants (age, time since stroke, type of stroke, etc.) and outcomes. In particular, we focused on any reported associations between fatigue and the following:

- sociodemographic variables (age, gender, ethnicity, educational level, living alone, marital status, social support, independency, employment, etc.),
- psychological factors (depressive symptoms, anxiety, coping style, quality of life, etc.),
- cognitive measures,
- clinical factors (type of stroke, time since stroke, infarct volume, stroke severity, infarct site, stroke etiology, imaging data, vascular risk factors, etc.),
- physical factors (walking activity, upper or lower limb function, aerobic fitness, etc.),
- blood laboratory tests,
- prestroke fatigue, sleeping disorders, and sleepiness,
- pain, appetite, and medications.

## 3. Results

Of the included articles, 96 were written in English, one was written in French and one was written in Korean. The included articles had been published between 1999 and 2015. The median number of included stroke patients was 100 (range: 9 to 3667). Twenty-seven studies assessed patients having suffered ischemic stroke or TIA, 6 assessed patients with hemorrhage, and 65 studied populations of both types of patients. The time since stroke ranged from the acute phase (less than 15 days after stroke) up to 2 years in 69 studies and was over 2 years in 24 studies. The time since stroke was not specified in 5 papers. Eighty-six of the 98 studies used at least one fatigue scale to evaluate fatigue level, with 4 applying a case definition of fatigue. Eleven studies employed a subsection of a health-related quality of life questionnaire to evaluate fatigue or vitality, and one study asked a single question about fatigue.

## 4. Sociodemographic Factors

Forty-six studies tested for associations between gender and PSF but 33 found no significant differences between males and females [5, 21, 23, 24, 29, 31–58]. Twelve showed a predominance of PSF in females [13, 20, 59–68], and only one study demonstrated a predominance of physical fatigue in males [12]. Similarly, 34 out of 44 studies failed to observe any association between PSF and age [5, 13, 23, 24, 29, 31–33, 35, 37, 40, 42–46, 49–58, 60, 61, 63–65, 67–69], whereas eight demonstrated a positive association [20, 22, 34, 36, 38, 47, 62, 66], and two demonstrated a negative association [21, 48]. Educational level (examined in 16 studies) was not associated with PSF [29, 42–44, 46, 48–52, 55, 57, 64, 65, 68, 69]. Ethnicity was not linked to PSF in two studies [32, 42] but was linked in a third study [36].

The impact of social factors on PSF has also been addressed in a few studies but the results remain to be confirmed. Eight studies failed to demonstrate a link between PSF and marital status [21, 23, 42, 43, 47, 55, 58, 64], and only one study found this type of association [62]. The authors of the latter study further postulated that PSF might be more frequent in patients living alone [62]. However, this result was not replicated in four other studies [36, 40, 48, 60]. Lack of social support was associated with more PSF in two studies [69, 70] but not in two larger studies [52, 55]. Lastly, one recent study showed a higher incidence of PSF in patients with dysfunctional familial relationships, patients with a lower family income, and patients living in rural areas [52]. PSF was not associated with family income in one study [69].

Twenty-five studies found that PSF was associated with greater disability and dependency [20, 22, 24, 25, 29, 32, 33, 36, 38, 43–45, 48, 52–54, 57, 60, 62, 64, 65, 68, 71–73], even though the association seems to be mediated by depression [74] and was not detected in 15 studies [5, 19, 21, 23, 31, 42, 46, 48–50, 56, 61, 67, 69, 75]. PSF was associated with less return to work (and particularly to a return to full-time work) soon or long after the stroke in seven studies [22, 29, 43, 44, 46, 76, 77] but not in another study [64].

Lastly, there are few reported associations between sociodemographic factors and PSF. Other than a trend towards more PSF in females and older patients (as seen for fatigue in the general population [78]), PSF does not seem to be related to educational level, ethnicity, marital status, or social support. Nevertheless, PSF is generally associated with disability, dependency, and infrequent return to work. Thus, PSF has a specific pattern of impact on patients' everyday lives.

## 5. Psychocognitive Factors

### 5.1. Emotional Disturbances

Fatigue is at least in part a subjective syndrome and depends on psychological factors such as stress linked to stroke itself, having a chronic disease and assuming the consequences of stroke in everyday life [10]. For many years, fatigue was considered to be a symptom of depression because the two conditions were often concomitant [14]; patients with depression are slower and more tired than nondepressed patients [79]. Indeed, patients often confuse fatigue and depression [7].

The great majority of studies (45 out of 48) found a correlation between PSF and poststroke depression [13, 18, 20–22, 24–26, 29, 31, 33, 35–38, 40, 41, 43, 44, 46–50, 52, 53, 57–59, 61–65, 67–69, 72, 75, 77, 80–84]. Hence, only three studies failed to observe this type of association [23, 55, 56]. The association has been studied at different time points after stroke: in the acute phase [64] and up to 24 months [46]. PSF is also associated with suicidality [85]. In the literature, between 29% and 34% of fatigued patients are depressed [5, 29, 47, 74]. Nonetheless, fatigue was observed in 14–50% of nondepressed patients [5, 21, 24, 29, 37, 61, 74], and 30% of fatigued patients did not have any anxious or depressive symptoms [24].

Depression and fatigue might be two separate processes, and the temporal relationship between the two is not well understood [86]. Depression might be a factor in the maintenance of fatigue over time [49]. It is noteworthy that a history of depression (i.e., before stroke) was not always controlled for in these studies. Although one study found that prestroke depression might be linked to PSF [20], another study did not [48].

Although anxiety has been less frequently assessed, it was associated with PSF in 13 studies [13, 22, 24, 26, 33, 35, 37, 44, 48, 57, 62, 63, 65] but not in two other studies [23, 55]. This link has been demonstrated from 1 to 18 months after stroke [22, 24, 48, 63].

The style of coping was also associated with the incidence of fatigue: PSF was more frequent in patients with emotion-oriented coping [38], passive coping, and external styles of coping [47, 57]. This result was not confirmed by another study [55].

The negative impact of PSF on quality of life has been demonstrated in 17 studies [19, 21, 32, 33, 40, 69, 77, 86–95].

Emotional factors (such as depression and anxiety) are particularly linked to PSF. At present, the relative influences of these syndromes on each other are not well understood. Nevertheless, we know that there is a clear link between these conditions. Studies of PSF must control for depressive and anxious symptoms as potential confounders. Additionally, PSF is clearly related to quality of life.

### 5.2. Cognitive Disorders

“Coping theory” states that fatigue is the result of compensatory efforts in response to demands following brain injury [14, 96, 97]. Neuropsychological impairments might contribute to the genesis of fatigue. In this context, the evaluation of cognitive disorders may help us to better understand PSF.

Eleven studies failed to observe correlations between fatigue questionnaire scores and the Mini Mental State Examination (MMSE) [98], even when studying different time points after stroke (from the acute phase to the longer term) [23, 32, 43, 44, 47–49, 63, 68, 75, 99]. One study found that the MMSE score was correlated with PSF but could not predict its change over time [33]. Moreover, this association disappeared when only nondepressed patients were considered, suggesting that cognitive impairment can be mediated by depression [33]. Nevertheless, we know that the MMSE (a measure of general cognitive functioning) is not really sensitive to poststroke attentional and executive disturbances and thus perhaps PSF [100]. The MoCA might provide a more sensitive evaluation of the potential relationships between cognition and PSF [101]. More extensive cognitive batteries might also be more informative.

Selective attention integrates mental, physical, and sensorial inputs when performing a task. Changes in selective attention lead to integration difficulties and thus greater efforts are required to compensate for this impairment [9]. Thus, attentional difficulties might be associated with PSF. Complaints of difficulty in concentrating were found to be associated with PSF up to one year after stroke [72]. One study found that fatigued and nonfatigued patients differed with regard to (i) sustained attention and alertness at 6 months and (ii) sustained attention, alertness, and divided attention at 12 months [22]. However, attentional performances were not related to PSF long after stroke [65]. Although a recent study demonstrated that fatigued and nonfatigued patients did not differ significantly in terms of reaction time [102], processing speed might be related to cognitive/mental fatigue [18, 103], physical fatigue [18], but not general fatigue [18]. Processing speed was correlated with PSF 3 and 6 months after stroke [46] and even up to 10 years after stroke [65].

Few studies evaluated PSF and executive functioning. The results did not show any correlation between fatigue scores and mental flexibility [46, 47, 103]. Although inhibition capabilities were correlated with PSF in nondepressed patients in one study [22], this result was not replicated in two other studies [46, 65]. Fluencies were also associated with fatigue (mainly cognitive fatigue) [18, 22], even though three studies failed to demonstrate a link with general fatigue [46, 65, 103].

Three studies have established a link between language abilities and PSF. One showed that aphasia was predictive of PSF in patients without prestroke fatigue [29]. It has also been demonstrated that patients with language disorders express more fatigue than patients without language disorders [62]. Nevertheless, when evaluating language with specific cognitive measures, the results depend on the time since stroke; one study found a significant association with language abilities 12 months after stroke but not 6 months after stroke [22]. However, the evaluation of aphasic patients is generally challenging, which explains why the potential links between language impairment and PSF are not well understood.

Few studies evaluated links between memory function and PSF. A recent study demonstrated a significant correlation between PSF 6 months after stroke and memory performance in a 10-word list-learning task [46]. However, the correlation was not significant 3 and 24 months after stroke. Another study evidenced similar fluctuations as a function of the time since stroke [22]. Immediate recall ability has been found to be correlated with (i) cognitive fatigue for verbal material and (ii) physical fatigue for visual memory [18]. In contrast, PSF was not correlated with delayed recall ability for verbal and visual memory [18, 65]. The results concerning working memory are also disparate, with two studies demonstrating an association [18, 65] and one study not demonstrating an association [103]. Further studies are required.

Other cognitive domains have not been extensively explored (visuoconstructive performances [65], visual neglect [29], orientation [21], and reasoning [46]) but do not appear to be associated with PSF.

PSF does not seem to be associated with general cognitive functioning. Some preliminary results demonstrated an association with attentional difficulties but need to be confirmed. At present, the data on executive functioning, memory, and language are too sparse to enable firm conclusions to be drawn.

## 6. Neurophysiological Factors

### 6.1. Neurological Factors

One can legitimately hypothesize that neurological disease factors in general (and stroke-associated parameters in particular) have an impact on PSF. However, few studies have evidenced links between PSF and stroke characteristics.

Ischemic and hemorrhagic strokes appeared to have much the same impact on fatigue in ten studies [5, 13, 23, 32, 35, 42, 47, 61–63]. However, the studies often focused on heterogeneous groups with a high proportion of ischemic stroke patients and thus a low proportion of hemorrhagic stroke patients; this might have influenced the statistical significance of the results. Infarct volume was not of importance in nine studies [29, 31, 33, 45, 46, 49, 50, 67, 68], and neither was thrombolysis [33]. At least one other previous stroke was significantly associated with PSF in four studies [21, 36, 62, 65] but not in 14 studies [20, 23, 31, 33, 44, 48–50, 55, 61, 63, 67, 68, 99]. Neither the time since stroke [5, 23, 25, 29, 42, 44, 51, 55, 56, 61, 65, 80] nor the stroke etiology [29, 31, 33, 34] was correlated with PSF in 12 and 4 studies, respectively.

According to two studies, fatigue was more frequent in patients with TIA [27, 37], suggesting that the presence of a lesion can influence PSF. Six studies found that PSF was associated with stroke severity [21, 22, 27, 29, 33, 38, 52], although the association was not significant in nondepressed patients [33]. Sixteen studies did not find this relationship [5, 20, 31, 35, 44, 46, 48–50, 53, 54, 60, 63, 65, 67, 68]. PSF was linked to long-term mortality rates in three studies [20, 43, 66].

Although PSF is more frequent after stroke than after TIAs, it appears that stroke characteristics (such as the type, severity, etiology, and infarct volume) are not predictive of PSF.

### 6.2. Imaging Data

In view of the literature data, one can hypothesize that fatigue is related to poor functional integration within the limbic system and the basal ganglia (associated with alterations in the frontothalamostriatal system) [104].

Stroke side was not linked to PSF in 17 studies [5, 13, 21–23, 35, 38, 39, 42, 44–48, 52, 55, 63]. In contrast, other studies found relationships between PSF and various stroke sites: posterior strokes [63], infratentorial lesions [48], and basilar infarcts [44]. However, most studies did not find any relationship between stroke site and PSF [13, 18, 20, 22, 29, 31, 33, 35, 57, 60, 65]. The lesion site might have a differential impact depending on the type of fatigue, with a trend towards more physical fatigue in patients with subcortical lesions and more cognitive fatigue in patients with cortical lesions [18].

Magnetic resonance imaging studies have investigated PSF. In line with the hypothetical involvement of subcorticofrontal systems in nondepressed patients, lesions located in the basal ganglia and internal capsule were found to be predictive of PSF [67], as were caudate infarcts [68]. PSF might also be linked with profound microbleeds [50].

The impact of white matter lesions has rarely been studied. The few available data suggest that fatigue is more frequent in patients with severe leukoaraiosis on a CT scan [20]. Nonetheless, four MRI studies failed to detect a link between white matter lesions and fatigue [33, 49, 50, 68]. However, a recent study of nondepressed patients demonstrated that white matter hyperintensities were not associated with PSF 3 months after stroke but were predictive of fatigue one year after stroke [49].

In summary, lesion side and site are not clearly associated with PSF. However, recent MRI studies have provided data that suggest the involvement of the subcorticofrontal system in fatigue.

### 6.3. Physical Deconditioning

Some recent studies have focused on physical deconditioning as a potential explanation for PSF. A fall in muscle strength might lead to an increase in the effort demand and thus greater fatigue. Thus, poor physical functioning might contribute to PSF [41, 64].

PSF was significantly related to walk scores (and particularly the number of steps) in four studies [23, 35, 42, 105] but not in three other studies [21, 56, 106]. PSF was not correlated with walking speed [56, 70, 107, 108] or upper or lower limb functioning [21, 23], except in one study [109]. Five studies observed correlations with balance, motor control and aerobic fitness [42, 70, 83, 110, 111], although six others did not demonstrate significant differences [23, 47, 55, 56, 70, 112]. However, this kind of measurement (gait, balance, etc.) might be more closely correlated with physical fatigue than with general or mental fatigue [18].

Recently, a study investigated the relationship between motor cortex excitability (measured following transcranial magnetic stimulation) and fatigue in stroke patients with minimal impairments [113]. Patients with high levels of fatigue exhibited higher motor thresholds, and those who perceived high physical efforts displayed low excitability of the inputs that drive motor cortex output. The researchers suggested that PSF might result from a difference between the effort produced by the patient and the actual motor output [113].

Physical deconditioning is a promising hypothesis in terms of rehabilitation: physical reconditioning therapies are based on the idea that maximizing activity and mobility could increase force and endurance and thus reduce PSF [86]. Physical fatigue scales and objective measurements (e.g., electromyography) are better indicators of fatigue related to physical condition but were not the focus of our review.

### 6.4. Biological Factors

To date, biological factors putatively involved in PSF have received little attention: this might nevertheless constitute a promising field in terms of finding pharmacological treatments.

Although some neuroendocrine hypotheses of PSF have been suggested [9, 104], cortisol, adrenocorticotropin hormone and thyroid hormone (T4, TSH) levels were not associated with PSF in one study [22].

Inflammatory hypotheses seem more promising but have not been extensively studied [9, 114]. In a pilot study, PSF was observed in patients with high levels of C-reactive protein [115]. However, this result was not replicated in a larger study [45]. In contrast, PSF was associated with high levels of interleukin- (IL-) 1*β* [45] and with low levels of IL-9 and the neuroprotective IL-1*β* antagonist IL-ra [45] in the poststroke acute phase, which was not the case with poststroke depression [81]. There was no correlation with other inflammatory agents (such as IL-8, IL-18, growth-related oncogene-*α*, IL-2, IL-4, IL-6, IL-10, IL-12, interferon-*γ*, and tumor necrosis factor-*α*) [45]. A recent study demonstrated a possible genetic contribution to PSF: particular single nucleotide polymorphisms in genes associated with immune response were possibly associated with susceptibility to or protection against PSF [31]. A proinflammatory response might be responsible for the development of fatigue short-term after stroke, which might then be complicated by psychosocial factors [45].

It has also been postulated that biochemical anomalies (such as vitamin B12 deficiency [116], low tryptophan levels, and high kynurenine levels [117]) might be associated with PSF. PSF was also found to be associated with glycemia [45, 53, 54], uric acid levels [53], and elevated homocysteine levels [54]. Levels of hemoglobin [45], total cholesterol, triglycerides, high-density lipoprotein, low-density lipoprotein, and fibrinogen were not correlated with PSF [54].

Hence, inflammatory factors might be associated with PSF. However, larger studies are needed to confirm the recently published preliminary results.

### 6.5. Comorbidities

Frequent comorbidities of stroke might also contribute to PSF [10]. Nevertheless, five questionnaire-based studies did not find an association between comorbidities and PSF [23, 29, 36, 47, 60].

#### 6.5.1. Vascular Risk Factors

Although vascular risk factors might conceivably be involved in PSF, the results tend to argue against this hypothesis. Three studies demonstrated that PSF was more frequent in patients with heart disease [20, 43, 52], whereas eight did not observe any difference [29, 31, 36, 44, 45, 53, 54, 61]. An association with diabetes was observed in three studies (all by the same research group) [20, 43, 44] but not in twelve others [29, 31, 33, 35–37, 49, 52–54, 61, 68]. Anemia was not correlated with PSF in one study [61]. An association with hyperlipidemia was found in one study [68] but not in four others [29, 31, 49, 52]. PSF was related to hypertension in one study [37] but not in fourteen others [20, 29, 31, 33, 35, 36, 44, 45, 49, 50, 53, 54, 68]. Smoking was associated with PSF in one study [29] but not in five others [31, 36, 43, 44, 52]. Alcohol consumption was also associated with PSF in one study [43] but not in three others [29, 46, 52]. Three studies found that body mass index was not correlated with PSF [20, 44, 52]. Migraine was correlated with PSF in one study only [44].

#### 6.5.2. Sleep Disorders

Sleep disorders are frequent after stroke, TIA [118–121], and subarachnoid hemorrhage [122] and might even constitute a risk factor for stroke [123, 124]. After a stroke, about 50% of patients complain of changes in their sleeping habits; they notably report sleeping longer at night and being more drowsy during the day [7, 125]. About 30% reported sleep disorders up to one year after stroke, with daytime sleepiness, longer sleep latency, and nonrefreshing sleep [47, 75]. Thus, PSF was associated with sleep disorders (assessed by questionnaires) in nine studies [20, 29, 49, 52, 64, 69, 75, 122, 126] but not in three others [47, 55, 61]. A correlation between PSF and daytime sleepiness was also observed in four studies [12, 35, 125, 126] but not in two others [36, 56].

#### 6.5.3. Prestroke Fatigue

Prestroke fatigue might also be associated with PSF. In a study of 220 stroke patients, 38% reported prestroke fatigue [29]. This factor was strongly associated with PSF in five studies [29, 33, 52, 64, 99], although 36% of the patients who did not suffer from preexisting fatigue also complained of PSF [29]. Two studies failed to demonstrate a link between pre- and poststroke fatigue [35, 63].

#### 6.5.4. Pain

Seven studies found a significant link between pain and PSF [20, 49, 56, 62, 69, 80, 127]. About 10% of patients displayed the triad of fatigue, depression, and pain, and about 20% suffered from fatigue and pain but not depression [20, 80]. Moreover, pain might be involved in the persistence of fatigue over time [49].

#### 6.5.5. Nutrition and Appetite

Nutrition contributes to PSF, since poor nutritional status was associated with low vitality [69, 128]. Fatigue has also been linked to a decrease in appetite [29].

#### 6.5.6. Medications

Lastly, medications taken to treat frequent comorbidities can also impact on PSF [9, 10]. Patients often report that their fatigue is due to medications [7]. Unfortunately, few studies have analyzed this potential influence. Three studies did not show any relationship between medications and PSF [29, 45, 46]. A further study found no association between PSF and beta-blockers or statins [61]. Other studies showed an association between PSF and the use of sedative drugs [52], antidepressants [33, 77, 80], hypnotics [20, 80], analgesics [20], and antihypertensive drugs [37].

In summary, PSF does not appear to be associated with vascular risk factors. In contrast, PSF is frequently associated with prestroke fatigue and poststroke sleeping disorders and daytime sleepiness. Pain, nutrition, and medications might also be linked to the presence of PSF, although further investigations are needed.

## 7. Conclusion

PSF is a frequent, disabling health condition that results from the complex interaction between the many factors reviewed here.

Although there is a trend towards a greater incidence of PSF in women and in elderly patients, sociodemographic factors (such as educational level, social support, and marital status) do not seem to be significantly associated with PSF. In contrast, psychological and life factors (such as depression, anxiety, and poor quality of life) are strongly linked to PSF. Even though studies evaluating overall cognitive function did not demonstrate correlations with fatigue scores, more extensive cognitive investigations revealed correlations with attentional performances in general and processing speed in particular.

Neurological factors (such as the type, severity, and etiology of stroke) and infarct volume do not appear to be associated with PSF. However, the presence of a lesion might be of importance because stroke patients are more fatigued than patients having suffered a TIA. Recent MRI data have suggested the involvement of the subcorticofrontal network in PSF. Biological data are rarely reported in studies of PSF. The inflammatory hypothesis is promising but must be confirmed in larger studies. Lastly, PSF appears to be independent of vascular risk factors but is associated with sleep disorders, prestroke fatigue, pain, and poor nutrition. The patient's medication might also be linked to the presence of PSF, although data on this subject are lacking.

A better understanding of PSF will enable healthcare workers to recognize this “invisible handicap” more frequently and explain it more clearly to their patients. Furthermore, a better understanding of PSF might facilitate the development of effective treatments strategies aiming at fatigue directly or indirectly through the treatment of associated factors.
